# Comorbidity prevalence and incidence in cancer survivors: a longitudinal All of Us study

**DOI:** 10.1093/jncics/pkaf093

**Published:** 2025-10-08

**Authors:** Jung Ae Lee, Ratna Pakpahan, Daniel J Amante, Ben S Gerber, Lin Yang

**Affiliations:** Department of Population and Quantitative Health Sciences, University of Massachusetts Chan Medical School, Worcester, MA, United States; Division of Public Health Sciences, Department of Surgery, Washington University School of Medicine, St Louis, MO, United States; Department of Population and Quantitative Health Sciences, University of Massachusetts Chan Medical School, Worcester, MA, United States; Department of Population and Quantitative Health Sciences, University of Massachusetts Chan Medical School, Worcester, MA, United States; Department of Cancer Epidemiology and Prevention Research, Cancer Care, Alberta, Canada

## Abstract

**Background:**

Comorbidities worsen cancer survival, but patterns of preexisting and new-onset comorbidities among cancer survivors are unknown.

**Methods:**

We investigated self-reported and clinically diagnosed comorbidity among cancer survivors in the All-of-Us program’s national database. Eight highly prevalent comorbidities were identified using self-reported data from the personal health history survey among cancer survivors (*n* = 20 534) and noncancer adults (*n* = 113 628) and validated among cancer survivors (*n* = 26 978) using data from electronic health records (EHRs). Among 5-year survivors (*n* = 9174) documented in EHR, we further estimated the incidence of new-onset comorbidities.

**Results:**

The most prevalent comorbidities identified in personal health history data were hypertension (40.5%), osteoarthritis (28.4%), depression (28.0%), and obesity (23.2%). EHR data identified preexisting comorbidities: hypertension (43.3%), osteoarthritis (29.4%), depression (19.4%), and obesity (19.1%). During 5-year survival, more than 50% of cancer survivors developed at least one new comorbidity, and more than 25% developed two or more. The onset of new comorbidities showed a sharp increase in the first-year postdiagnosis. Incidence rates varied by age, race, and ethnicity.

**Conclusion:**

Future research is needed to develop effective strategies to prevent new-onset comorbidities during and after cancer treatment.

## Introduction

In developed countries, aging and improved cancer survival owing to advancements in early detection and treatment have resulted in a rapidly growing cancer survivor population.^1^ In the United States, 18.1 million individuals are currently living with or after cancer, and this number is expected to reach 21.6 million by 2030.^2^^,^^3^ Approximately two-thirds of US cancer survivors are aged older than 65 years, many of whom present with age-related comorbidities.^4^^,^^5^ Comorbidities bear substantial excess out-of-pocket costs and pose a serious burden to the health-care system.^5^ Additionally, they impair quality of life and shorten survival from many types of cancer including prostate, lung, breast, digestive system, gynecological, urinary system, and head and neck.^6^

Cancer treatment can exacerbate existing comorbidities and increase the risk of new comorbidities.^5^^,^^7-9^ Accelerated aging in cancer survivors likely elevates the risk of comorbidities that affects older and younger survivors,^5^^,^^8^ posing unique late effects during cancer survivorship.^7^^,^^9^ Although a high prevalence of comorbidities in cancer survivors has been documented,^10^ most studies focused solely on post-treatment survivors. Given the known treatment-induced accelerated aging, cancer survivors may develop new comorbidities with a different trajectory.^11^ Current literature is limited to distinguish whether the comorbidity among cancer survivors was preexisting at diagnosis or newly developed after diagnosis and cancer treatment, which hinders the development of targeted interventions.

To date, the natural history of new-onset comorbidities in cancer survivors is largely unknown. Furthermore, it is unclear whether these comorbidities differ by cancer type, age, race, and ethnicity. These knowledge gaps hinder the identification of high-risk cancer survivors for better screening, early detection, and timely implementation of chronic condition management strategies. In the present study, we estimated the pre-diagnostic prevalence and the post-diagnostic incidence of chronic conditions in a large sample of US cancer survivors and examined high-risk subpopulations by cancer type, age group, and racial/ethnic background.

## Methods

### Study population

All of Us is a National Institutes of Health–funded initiative to advance precision medicine and improve health by enrolling more than 1 million individuals across the United States in a prospective cohort study.^12^ Participants are recruited through many participating health-care organizations and in partnership with Federally Qualified Health Centers to engage underrepresented populations. All participants provided informed consent digitally through an online platform (joinallofus.org). Our analyses included data from the Controlled Tier Dataset v6, collected between May 2018 and January 2022. We accessed the personal and family health history survey and participant-shared electronic health records (EHRs) to analyze self-reported and clinically documented chronic conditions. Analyses were conducted in accordance with the All of Us Data User Code of Conduct and the UMass Chan institutional review board.

### Self-reported cancer diagnosis and chronic conditions

We conducted cross-sectional analyses using personal health history data and compared the prevalence of self-reported common chronic conditions between noncancer adults and cancer survivors. Cancer survivors were defined as individuals who self-reported any cancer (except for skin cancer) diagnosis in responding to the question (question concept ID = 43528760): “Has a doctor or healthcare provider had or has ever diagnosed you with any specified cancers? Select all that apply.” We captured the 20 reported cancer types. Those who responded with “no cancer diagnosis” were considered noncancer adults. Approximately 11.8% of cancer survivors had a second cancer diagnosis and were also included in the comorbidity analyses as “2 or more cancers” (see Figure S1 for sample flow chart).

### Health records detected cancer diagnosis and chronic conditions

We conducted longitudinal analyses using EHR data to estimate the prevalence of preexisting chronic conditions and the incidence of new-onset chronic conditions, including before (or at) and after cancer diagnosis. We captured 10 cancer types: breast, prostate, blood, colon and/or rectal, lung, thyroid, endometrial, kidney, bladder, and ovarian cancer. We used the first cancer type for individuals with multiple cancers (17%). All cancer diagnoses, chronic condition diagnoses, and time stamps of diagnoses were identified by standard concept names (Table S1). We sorted the chronic condition diagnosis dates chronologically in relation to the date of first cancer diagnosis to differentiate preexisting and new-onset chronic conditions (see Figure S2 for sample flow chart).

### Covariates

Date of birth, sex assigned at birth, race, and ethnicity data were obtained from the demographics prepackaged concept set within the All of Us workspace. Age at the time of first cancer diagnosis was calculated from the date of birth and categorized into 18-39, 40-54, 55-64, 65-74, and 75-100 years. Sex at birth was defined as female and male. Race was categorized into Asian, Black, Remaining (including Middle Eastern or North African, Native Hawaiian or other Pacific Islander, and more than one race), and White. Ethnicity was categorized into Hispanic or Latino vs not Hispanic or Latino.

## Statistical analysis

Three stages of analyses were conducted. First, we identified the most prevalent chronic conditions self-reported in personal health history. The prevalence was calculated among noncancer adults and cancer survivors overall and by cancer type (including the diagnosis of a second cancer). These analyses identified the 8 most common chronic conditions: hypertension, osteoarthritis, neuropathy, depression, obesity, type 2 diabetes, sleep apnea, and kidney stones, based on their relevancy to the major human health systems (skeletal muscle system, endocrine system, circulatory system, etc.) (Table S2).

Second, we analyzed EHR data limited to cancer survivors to describe the prevalence and incidence of preexisting and new-onset chronic conditions, respectively. For preexisting chronic conditions, the prevalence of each of the 8 conditions was calculated among all EHR-identified cancer survivors, overall and by cancer type. We calculated the annual proportions of new-onset chronic conditions in a subset of cancer survivors who had at least 5 years of follow-up records available. This ensures that our findings are relevant to long-term survivorship.

Third, we calculated the incidence rate of new-onset comorbidity among cancer survivors who had at least 5 years of follow-up records in the EHR. We conducted cancer-specific quasi-Poisson regressions to estimate the 5-year incidence rates for the number of chronic conditions (0-8), adjusting for age, sex, race, and the number of preexisting comorbidities. Analyses for sex-specific cancers such as breast, prostate, endometrial, and ovarian cancers were not adjusted for sex. The overdispersion of count data was handled with corrected standard error using the quasi-model. We reported the adjusted incidence rate ratio among cancer survivors of Asian, Black, and Remaining race using White race as the reference. To address multiple testing, *P* values were adjusted by the Dunnett method, which is effective for the baseline comparison.^13^ Missing data (13.4% for race and 2.8% for sex) were included in the analysis as a category. We performed sensitivity analyses using multiple imputed datasets, generated through chained equations with predictive mean matching.^14^

All statistical analyses used a significance level of 0.05 with 2-sided testing and were conducted within the Jupyter Notebook environment of the All of Us Workbench, utilizing the R programming language (R Core Team, 2024, Version 4.4.0, Vienna, Austria).

## Results

The sample sizes were 20 534 cancer survivors (vs 113 628 noncancer adults) self-reported in personal health history, 26 978 cancer survivors identified in EHR with available data on preexisting chronic conditions, and 9174 cancer survivors identified in EHR with available data on preexisting and new-onset chronic conditions. The overlap of cancer survivors between personal health history and EHR was 10 098, representing 37.4% of EHR’s survivors. The study sample flowchart is depicted in Figures S1-S2. Table 1 summarizes the study population characteristics in personal health history and EHR, respectively.

**Table 1.** The characteristics of the study populations of the personal health history and EHRs in the All-of-Us program

Sociodemographic characteristics	Personal health history self-reported cancer diagnosis and comorbidities				EHR clinically diagnosed cancer and comorbidities			
	Noncancer adults		Cancer survivors		Cancer survivors		Cancer survivors 5 or more years	
	(*n* = 113 628)		(*n* = 20 534)^a^		(*n* = 26 978)^b^		(*n* = 9174)^c^	
	% of n	Mean (SD)^d^	% of n	Mean (SD)	% of n	Mean (SD)	% of n	Mean (SD)
Age, y								
18-39	29.83	0.84 (1.0)	5.54	1.27 (1.2)	8.14	2.42 (2.0)	7.39	2.86 (2)
40-54	22.43	1.48 (1.5)	14.94	1.68 (1.6)	24.53	3.08 (2.1)	27.55	3.38 (2)
55-64	20.53	1.72 (1.6)	23.26	1.85 (1.6)	30.07	3.28 (2.0)	33.97	3.53 (1.9)
65-74	20.25	1.8 (1.5)	36.54	1.88 (1.5)	27.76	3.29 (1.9)	25.59	3.61 (1.8)
75 and older	6.95	1.66 (1.3)	19.72	1.69 (1.4)	9.49	3.19 (1.7)	5.50	3.53 (1.6)
Sex at birth								
Female	63.76	1.4 (1.4)	60.22	1.80 (1.5)	59.62	3.15 (2.0)	59.83	3.4 (1.9)
Male	32.17	1.41 (1.4)	35.40	1.70 (1.4)	37.55	3.16 (2.0)	37.33	3.55 (1.9)
Missing or intersex	4.07	1.61 (1.5)	4.38	1.94 (1.5)	2.84	3.23 (2.0)	2.82	3.42 (1.9)
Race								
Asian	3.63	0.64 (1.0)	1.61	1.17 (1.2)	1.92	2.20 (1.7)	1.56	2.52 (1.6)
Black	8.83	1.58 (1.4)	6.09	1.95 (1.5)	13.83	3.67 (2.0)	11.21	4.14 (1.8)
Remaining^e^	2.67	1.16 (1.3)	1.65	1.79 (1.5)	1.78	2.98 (2.0)	1.68	3.56 (2)
White	70.55	1.47 (1.4)	79.84	1.77 (1.5)	66.46	3.03 (2.0)	72.16	3.31 (1.9)
Missing	14.32	1.28 (1.4)	10.82	1.75 (1.5)	16.0	3.35 (2.0)	13.4	3.75(1.9)
Ethnicity								
Hispanic or Latino	11.12	1.11 (1.3)	5.90	1.62 (1.4)	12.70	3.36 (2.1)	9.98	3.84 (1.9)
Not Hispanic or Latino	83.44	1.44 (1.4)	87.88	1.78 (1.5)	82.75	3.12 (2.0)	85.61	3.41 (1.9)
Missing	5.45	1.61 (1.5)	6.22	1.88 (1.5)	4.55	3.21 (2.0)	4.40	3.47 (1.9)

Abbreviation: EHR = electronic health record.

a This cohort includes individuals who responded “yes” to any of the 20 cancer questions in personal health history.

b This cohort includes participants with any record of 10 cancers in EHR.

c This cohort includes participants with any record of 10 cancers in EHR, with 5 or more years of follow-up records.

d Mean (SD) of the number of comorbidities: hypertension, osteoarthritis, neuropathy, depression, obesity, type 2 diabetes, sleep apnea, and kidney stones.

e Remaining category includes Middle Eastern or North African, Native Hawaiian or Other Pacific Islander, and more than one population.

### Prevalence of self-reported comorbidities

Table 2 represents the prevalence of self-reported chronic conditions among noncancer adults and cancer survivors by 20 cancer types identified in personal health history. Overall, 11.8% (range = 13.6%-49.6%) of cancer survivors had a second cancer diagnosis, along with the following highly prevalent chronic conditions: hypertension (40.5%, range = 29.0%-54.5%), osteoarthritis (28.4%, range = 18.2%-40.0%), neuropathy (13.1%, range = 9.7%-20.9%), depression (28%, range = 17.5%-46.4%), obesity (23.2%, range = 14.0%-42.0%), type 2 diabetes (12.7%, range = 7.2%-26.9%), sleep apnea (20.1%, range = 15.4%-29.8%), and kidney stones (11.3%, range = 7.6%-17.9%) across various cancer types. Compared with noncancer adults, cancer survivors reported a higher number of comorbidities (Mean 1.77 [SD 1.49] vs 1.41 [1.43]; Mann-Whitney test, *P* < .001).

**Table 2.** Summary of self-reported comorbidity prevalence by cancer type among cancer survivors (*n* = 20 534), assessed from the personal health history in the All of Us program

Cancer diagnosis and type	Sample size	2 or more cancers, %	Hypertension, %	Osteoarthritis, %	Neuropathy, %	Depression, %	Obesity,%	Type 2 diabetes, %	Sleep apnea, %	Kidney stones, %
Noncancer adults^a^	113 628	NA	29.8	18.4	5.9	31.1	23.3	9.0	15.4	8.4
Breast	6002	13.6	35.6	34.6	13.0	29.2	23.5	10.0	16.0	7.6
Prostate	3442	14.8	50.9	21.4	9.7	17.5	14.0	13.7	25.5	15.3
Blood	1732	22.5	37.6	27.4	19.4	26.6	20.2	12.2	20.4	11.5
Cervical	1472	22.1	34.9	32.1	14.3	46.4	33.6	13.5	18.5	11.8
Thyroid	1397	27.7	40.2	28.0	10.5	31.6	33.1	14.5	21.5	11.9
Colon rectal	1177	27.7	46.6	26.1	18.2	26.7	22.9	16.5	20.0	11.4
Kidney	775	32.9	54.5	24.8	11.9	27.1	29.4	22.1	29.8	17.9
Bladder	767	33.0	46.5	26.2	12.0	19.2	20.3	15.4	21.6	16.4
Lung	759	34.9	45.3	29.9	13.4	27.8	19.1	14.1	19.1	10.5
Endometrial	662	30.8	45.3	40.0	15.0	37.8	42.0	16.2	22.4	10.6
Ovarian	546	35.2	36.3	32.2	20.0	38.5	32.2	10.6	18.1	10.1
Head/neck	520	30.4	41.3	24.6	14.6	27.1	21.3	13.1	19.6	10.8
Bone	335	49.6	37.0	21.8	20.9	29.0	20.3	16.1	16.7	13.7
Brain	286	31.5	29.0	18.2	15.0	39.2	21.3	7.7	15.4	8.7
Endocrine	202	45.5	46.0	27.7	13.4	34.2	23.8	19.8	16.8	17.3
Pancreatic	186	30.6	39.2	24.2	16.7	22.0	19.9	26.9	17.2	13.4
Esophageal	166	37.3	45.8	21.7	16.3	26.5	19.3	14.5	25.3	15.1
Stomach	133	46.6	>48.0	27.8	<15.0	36.8	29.3	21.8	28.6	16.5
Eye	111	32.4	31.5	27.9	<18.0	40.5	25.2	<18.0	20.7	<18.0
Other	2709	22.6	39.1	27.7	15.2	28.4	23.2	13.6	20.7	12.2
Missing rate	NA	NA	2.8	2.8	4.1	3.2	22.7	3.4	2.7	2.6

a Noncancer adults’ prevalence is included for comparison.

Note 1: The sample sizes over cancer type sum to 23 397, not 20 534, because of 11.8% having multiple cancers.

Note 2: Overall, hypertension (40.5%), osteoarthritis (28.4%), neuropathy (13.1%), depression (28%), obesity (23.2%), type 2 diabetes (12.7%), sleep apnea (20.1%), and kidney stones (11.3%).

Note 3: According to the All of Us program’s dissemination policy, some numbers are not exactly shown in the table and are replaced with “<” or “>” to prevent potential identification of participants, such as for sample sizes less than 20.

### Prevalence of clinically diagnosed preexisting comorbidities

Table 3 summarizes the prevalence of clinically diagnosed preexisting comorbidity conditions by cancer type identified in EHRs. The chronic disease prevalences were hypertension (43.3%, range = 35.5%-56.8%), osteoarthritis (29.4%, range = 26.7%-39%), neuropathy (23.1%, range = 20.1%-32.7%), depression (19.4%, range = 13.5%-29.7%), obesity (19.1%, range = 14.8%-28.9%), type 2 diabetes (15.9%, range = 10.0%-26.5%), sleep apnea (11.8%, range = 8.3%-17.4%), and kidney stones (5.3%, range = 3.2%-12.5%). Across all cancer types, 64.2% (range = 56.9%-77.8%) of cancer survivors had at least 1 of 8 highly prevalent comorbidities at the time of cancer diagnosis.

**Table 3.** Prevalence (%) of clinically diagnosed preexisting comorbidity at diagnosis by cancer type from the electronic health record data (*n* = 26 978)^a^

Cancer type	Sample size	Hypertension, %	Osteoarthritis, %	Neuropathy,^b^ %	Depression, %	Obesity, %	Type 2 diabetes, %	Sleep apnea, %	Kidney stones, %	Any of 8 conditions, %
Breast	7759	35.5	27.3	20.1	19.3	17.2	12.0	8.3	3.2	56.9
Prostate	4838	50.2	30.6	21.7	13.5	14.8	14.9	14.4	6.8	67.9
Blood	4535	41.3	27.4	24.1	18.3	18.1	16.9	11.6	4.8	61.9
Colon rectal	2228	48.3	31.2	25.1	22.6	21.1	20.2	11.2	5.8	68.8
Lung	1436	56.8	39.0	32.7	29.7	20.7	21.9	17.4	6.3	77.8
Thyroid	1722	37.0	27.5	24.6	20.7	22.8	15.2	11.4	4.2	62.0
Endometrial	1538	42.2	28.6	22.0	23.3	28.9	18.0	12.2	4.0	65.9
Kidney	1205	56.3	32.0	25.1	21.5	27.8	26.5	17.3	10.8	75.8
Bladder	975	52.6	34.6	28.7	20.3	19.0	18.5	15.9	12.5	72.0
Ovarian	742	35.6	26.7	22.1	22.0	20.1	10.0	8.9	4.0	61.6
Overall	26978	43.3	29.4	23.1	19.4	19.1	15.9	11.8	5.3	64.2

a Each individual was counted once by their first cancer type.

b Neuropathy is defined using SNOMED CT code 386033004 (Systematized Nomenclature of Medicine – Clinical Terms), a broad term encompassing peripheral neuropathy and polyneuropathy. Subtype-specific analyses are presented in Table S6.

### Annual proportion of comorbidities among long-term survivors

**Figure 1.**
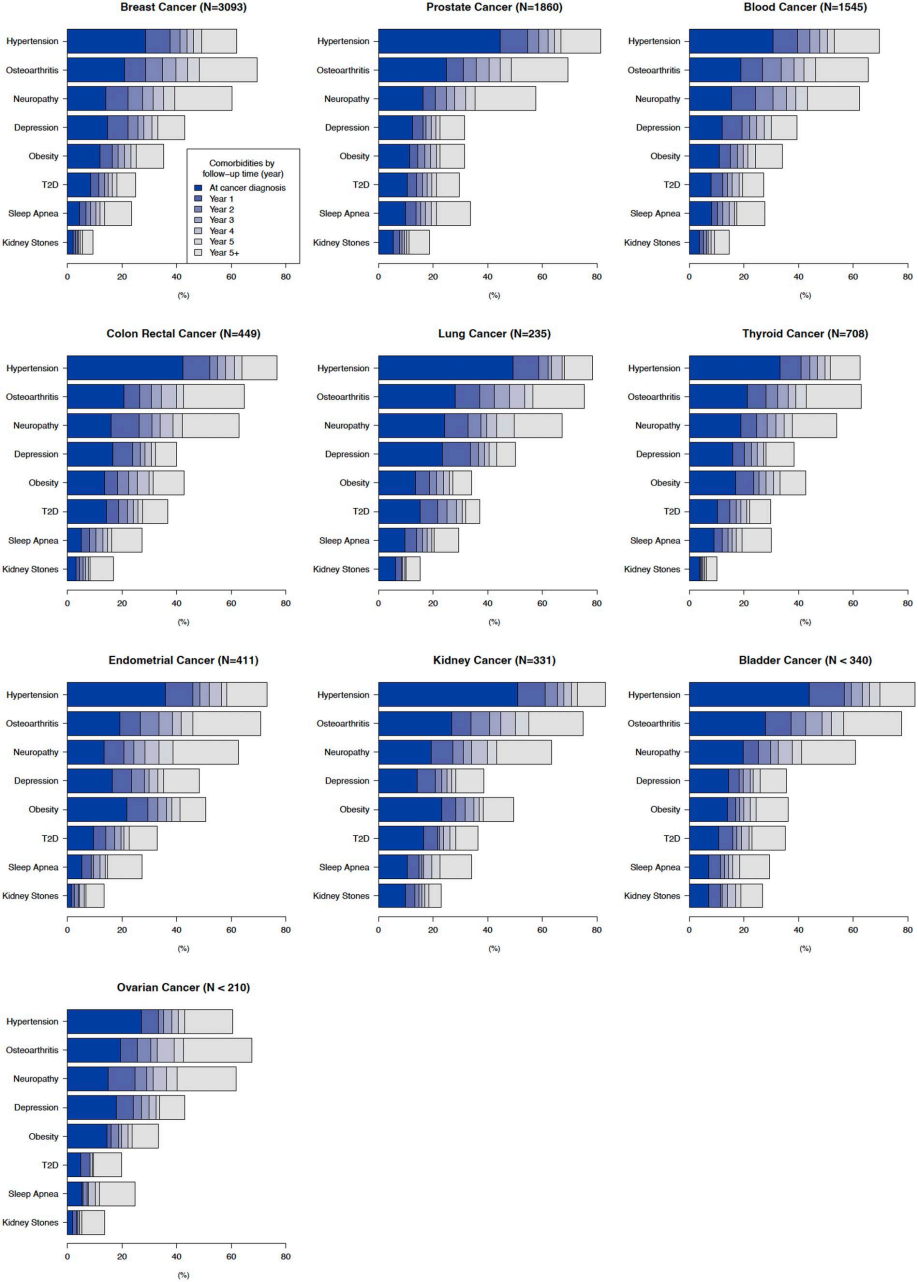
The annual proportion of clinically diagnosed new-onset comorbidities by cancer type, based on electronic health records from cancer survivors with at least 5 years of follow-up (*n* = 9174). For each cancer type, the incidence of 8 different comorbidities is reported at cancer diagnosis and yearly intervals from 1 to 5 years after the cancer diagnosis. Each year’s portion is labeled as follows: Year 1 for 0-1 year, Year 2 for 1-2 years, Year 3 for 2-3 years, Year 4 for 3-4 years, Year 5 for 4-5 years, and Year 5+ for more than 5 years, representing the new-onset comorbidity following the cancer diagnosis. Abbreviation: T2D = type 2 diabetes.

Figure 1 illustrates the prevalence of comorbidities at the time of cancer diagnosis and annual proportion of new-onset comorbidities from 1 to 5 years postcancer diagnosis among 9174 survivors identified in EHRs. Comparing with all cancer survivors identified in EHRs (*n* = 26 978), survivors with at least 5-year follow-up data (*n* = 9174) were younger and more likely to be White and non-Hispanic (Table S3). Across all cancer types, the annual proportion of each new-onset comorbidity was the highest within the first year of postdiagnosis and then stabilized in subsequent years.

### Age, racial, and ethnic disparities in new onset comorbidity incidence

Multivariable adjusted quasi-Poisson regressions revealed statistically significant disparities in the number of chronic conditions (0-8) across age groups in several cancer types. Age had a linear association with incidence of new-onset comorbidities among breast and prostate cancer survivors (Table 4). Breast cancer survivors aged 55-64, 65-74, and 75 years and older developed 15%, 40%, and 42% more chronic conditions, respectively, within 5 years than those at ages 18-54 years (*P* < .05). Similarly, prostate cancer survivors in these age groups developed 28%, 35%, and 46% more conditions, respectively. In certain cancers, the age effect was nonlinear. The colon rectal cancer survivors aged 65-74 years had the highest comorbidities among the age groups, with an incidence rate ratio of 1.4 (95% CI = 1.05 to 1.87). Similary, thyroid cancer survivors aged 65-74 years showed 1.64 (95% CI = 1.21 to 2.23). However, for both cancers, the age effect after 75 years (vs 18-54 years) no longer existed.

**Table 4.** Quasi-Poisson regression models estimated incidence rate ratio^a^ for the number of new-onset comorbidities during the 5 years of survival (*n* = 9174) by age and race in each cancer type^b^

Cancer type	Mean (SD) Q2 and Q3^c^	Age, y	Incidence rate ratio (reference 18-54 y)	95% CI	*P* ^d^	Race	Incidence rate ratio (reference White)	95% CI	*P* ^d^
Breast (*n* = 3093)	1.3 (1.4)	55-64	1.15	(1.03 to 1.27)	.007	Asian	0.94	(0.68 to 1.30)	.934
	Q2 = 1,^e^ Q3 = 2^f^	65-74	1.40	(1.25 to 1.58)	<.001	Black	1.59	(1.39 to 1.82)	<.001
		75 and older	1.42	(1.12 to 1.79)	.002	Remaining^g^	1.48	(1.08 to 2.03)	.009
Prostate (*n* = 1860)	1.2 (1.2)	55-64	1.28	(1.04 to 1.57)	.016	Asian	1.25	(0.58 to 2.70)	.84
	Q2 = 1, Q3 = 2	65-74	1.35	(1.09 to 1.66)	.002	Black	1.33	(1.11 to 1.59)	<.001
		75 and older	1.46	(1.12 to 1.91)	.002	Remaining^g^	1.45	(0.95 to 2.23)	.11
Blood (*n* = 1545)	1.4 (1.4)	55-64	0.95	(0.82 to 1.10)	.71	Asian	1.13	(0.64 to 1.99)	.90
	Q2 = 1, Q3 = 2	65-74	1.17	(1.00 to 1.38)	.05	Black	1.43	(1.19 to 1.72)	<.001
		75 and older	1.12	(0.83 to 1.51)	.69	Remaining^g^	1.17	(0.71 to 1.93)	.80
Colon rectal (*n* = 449)	1.3 (1.4)	55-64	1.17	(0.89 to 1.54)	.39	Asian	0.90	(0.31 to 2.61)	>.99
	Q2 = 1, Q3 = 2	65-74	1.40	(1.05 to 1.87)	.02	Black	1.13	(0.78 to 1.65)	.80
		75 and older	1.07	(0.66 to 1.73)	.96	Remaining^g^	1.19	(0.37 to 3.79)	>.99
Lung (*n* = 235)	1.4 (1.4)	55-64	1.24	(0.80 to 1.92)	.50	Asian	0.45	(0.08 to 2.39)	.60
	Q2 = 1, Q3 = 2	65-74	1.20	(0.77 to 1.86)	.60	Black	1.27	(0.83 to 1.96)	.40
		75 and older	1.04	(0.50 to 2.13)	>.99	Remaining^g^	1.27	(0.47 to 3.38)	.90
Thyroid (*n* = 708)	1.1 (1.3)	55-64	1.28	(1.02 to 1.62)	.03	Asian	0.74	(0.32 to 1.68)	.74
	Q2 = 1, Q3 = 2	65-74	1.64	(1.21 to 2.23)	<.001	Black	1.69	(1.20 to 2.38)	<.001
		75 and older	1.15	(0.54 to 2.42)	.92	Remaining^g^	1.11	(0.56 to 2.20)	.97
Endometrial (*n* = 411)	1.4 (1.3)	55-64	1.17	(0.92 to 1.50)	.29	Asian	2.24	(1.02 to 4.92)	.04
	Q2 = 1, Q3 = 2	65-74	0.87	(0.64 to 1.20)	.61	Black	1.50	(1.10 to 2.05)	.005
		75 and older	1.69	(0.97 to 2.96)	.07	Remaining^g^	1.29	(0.43 to 3.87)	.91
Kidney (*n* = 331)	1.4 (1.4)	55-64	0.94	(0.70 to 1.27)	.90	Asian	1.01	(0.21 to 4.88)	>.99
	Q2 = 1, Q3 = 2	65-74	1.00	(0.72 to 1.40)	>.99	Black	1.43	(0.99 to 2.06)	.06
		75 and older	1.17	(0.69 to 2.00)	.80	Remaining^g^	1.78	(0.94 to 3.37)	.09
Bladder (*n* < 340)	1.3 (1.3)	55-64	1.40	(0.96 to 2.06)	.09	Asian	0.64	(0.14 to 2.98)	.80
	Q2 = 1, Q3 = 2	65-74	1.32	(0.91 to 1.94)	.20	Black	1.57	(0.91 to 2.70)	.10
		75 and older	1.41	(0.90 to 2.22)	.17	Remaining^g^	1.07	(0.44 to 2.64)	>.99
Ovarian (*n* < 210)	1.0 (1.1)	55-64	1.00	(0.67 to 1.50)	>99	Asian	1.02	(0.26 to 4.06)	>.99
	Q2 = 1, Q3 = 2	65-74	1.28	(0.77 to 2.14)	.50	Black	1.59	(0.81 to 3.10)	.30
		75 and older	0.60	(0.09 to 3.84)	.80	Remaining^g^	0.54	(0.08 to 3.67)	.80

Abbreviation: CI = confidence interval.

a Incidence rate ratio compares the rate at which new disease cases occur in one group with the rate in another group over a specified period.

b Quasi-Poisson regression models provide the estimated incidence rate ratios adjusted for age, race, sex, and the number of preexisting comorbidities.

c Mean (SD), Q2 = second quartile (median), and Q3  = third quartile (75th percentile) of the number of new comorbidities during 5 years of survival after the cancer diagnosis.

d *P* values are adjusted by the Dunnett method to address the multiple testing problem.

e Q2 = 1 indicates that more than 50% of survivors have experienced at least one comorbidity for 5 years.

f Q3 = 2 indicates that more than 25% of survivors have experienced at least two comorbidities for 5 years.

g Remaining category includes Middle Eastern or North African, Native Hawaiian or Other Pacific Islander, and more than one population.

With respect to race and ethnicity, the adjusted 5-year incidence rate ratios and 95% confidence intervals for Black (vs White) cancer survivors were 1.59 (95% CI = 1.39 to 1.82) for breast, 1.33 (95% CI = 1.11 to 1.59) for prostate, 1.43 (95% CI = 1.19 to 1.72) for blood, 1.69 (95% CI = 1.20 to 2.38) for thyroid, and 1.50 (95% CI = 1.10 to 2.05) for endometrial cancers (Table 4). Additionally, breast cancer survivors of Remaining race had 48% more chronic conditions than those of White race. The results remained consistent with missing data imputations (Table S4). Because of the limited sample sizes in race and ethnicity categories, the effect of ethnicity was investigated separately by replacing the race variable in the regression model. The adjusted 5-year incidence rate ratios for Hispanic or Latino (vs not Hispanic or Latino) cancer survivors were 1.43 (95% CI = 1.27 to 1.62) for breast, 1.31 (95% CI = 1.06 to 1.63) for prostate, 1.42 (95% CI = 1.20 to 1.67) for blood, 1.63 (95% CI = 1.25 to 2.12) for colon/rectal, and 1.32 (95% CI = 1.00 to 1.74) for endometrial cancers (Table S5).

## Discussion

To our knowledge, this study is the first to comprehensively investigate the burden of comorbidities in a large, diverse sample of cancer survivors using a longitudinal study design. Hypertension, osteoarthritis, neuropathy, depression, obesity, type 2 diabetes, sleep apnea, and kidney stones were the most commonly diagnosed comorbidities among cancer survivors. At cancer diagnosis, hypertension affected 43.3% of cancer survivors, whereas a history of kidney stone affected 5.3% of cancer survivors, with varying prevalence across cancer types. Postcancer diagnosis, new-onset comorbidities increased rapidly within first year, affecting most 5-year survivors of all cancer types.

Our findings support and extend evidence from previous studies on chronic conditions in the US cancer survivor population.^10^^,^^15^^,^^16^ Early studies revealed that cancer survivors experience a higher burden of comorbidities and symptoms compared with those without cancer. A 2007 study reported that pain, insomnia, and psychosocial distress were experienced among 34%, 30%, and 26% of 1904 cancer survivors, respectively, which were statistically significantly higher compared with noncancer participants.^15^ A recent survey of 7565 cancer survivors between 2010 and 2017 reported a similar, higher prevalence of chronic pain (30.8%) compared with noncancer controls.^16^ Furthermore, multimorbidity^17^ (multiple co-occurring chronic conditions) is increasing over time in cancer survivors. In one longitudinal study, the prevalence of multiple chronic conditions (3 or more) among cancer survivors increased from 43.7% to 46.5% from 2002 to 2018. This translates to an estimated number of cancer survivors living with at least 3 comorbidities rising from 4.7 million to 8.1 million in the United States.^10^

To our knowledge, there is little data available to describe the burden and pattern of new-onset comorbidities postcancer treatment. Our work uniquely leverages the multimodal data in All of Us combining survey and EHR data, with time stamps available to separate pre- and postcancer comorbidities. This provides population-level evidence on treatment-induced comorbidity, as reflected in the abruptly increasing chronic disease incidence shortly after diagnosis. It also draws attention to other potential psychologic and physiologic changes that increase risk of comorbidity, such as chronic inflammation, immune dysregulation, stress, or maladaptive behavior change. The recognition of comorbidity due to treatment-related toxicity and these risks support the need for better survivorship care.^18^

The effect of age on cancer comorbidities is complex. Cancer is an age-related disease. In our analysis, approximately 67% of cancer occurs in individuals aged older than 55 years. The age effect on the number of postcancer comorbidities is linear in breast and prostate cancer but not clear in other cancer types (eg, thyroid) after controlling for race. The plateau in risk of mortality and chronic conditions for survivors aged older than 75 years is found in the literature.^19^^,^^20^ These older adults may experience less screening or investigation into new conditions, or this may demonstrate survival bias.

Racial or ethnicity disparity is noted in breast, prostate, blood, colon rectal, thyroid, endometrial, bladder, or kidney cancers in the number of comorbidities within the first 5 years of survivorship, potentially contributing to the gap in mortality.^21-23^ Future research should consider whether those from racial minority groups are born in or outside the United States, which also experiences disparities. These often reflect lifestyle-related differences such as smoking, obesity, and alcohol use.

Cancer survivors face elevated risk of treatment-related comorbidities. Our findings show that first-year prevalence is substantially higher than in subsequent years across all chronic conditions. Contributing factors vary by treatment: chemotherapy can cause neuropathy;^24^ radiation therapy may damage surrounding organs and tissues, such as the heart and kidneys, and increase the risk of hypertension and kidney stones;^25^ and hormone therapies may induce metabolic syndrome,^26^ leading to diabetes and obesity. Cancer-related depression, fatigue, and sleep disturbance,^27^ along with reduced physical activity,^28^ further compound comorbidity burden.

Over the years, cancer survival has improved substantially, with 69.2% of patients diagnosed in 2014-2020 surviving at least 5 years.^29^ As the survivor population grows, cancer survivorship care has emerged as a pressing public health issue.^30^ Highly prevalent comorbidities impair their quality of life and increase mortality risk.^31^^,^^32^ Individualized chronic disease risk assessment tools and improved screening practice are urgently needed to improve cancer survivorship. Lifestyle interventions, such as diet and physical activity, have been recommended posttreatment.^33^^,^^34^ It remains unclear whether early intervention, initiated before or during cancer treatment, can effectively prevent new-onset comorbidities among cancer survivors.

Our study has several strengths. We leveraged data from the All of Us Research Program, a demographically diverse cohort containing multimodal data (survey and EHR). A key strength is the temporal characterization of comorbidities before and after a cancer diagnosis. This type of longitudinal data offers potential pathways for more precise care of individual conditions at diagnosis, during treatment, and by cancer type and demographic factor(s). Another major strength of our study is the comparison of comorbidities from self-reported (personal health history) and EHR data. This showed good alignments for the majority of conditions, with notable discrepancies observed for neuropathy. In personal health history data, self-reported neuropathy was more likely to reflect such conditions associated with cancer treatment. Meanwhile, neuropathy was defined broadly in the EHR, encompassing peripheral nerve disorders and polyneuropathy (detailed in Table S6). This captures chemotherapy-induced peripheral neuropathy as well as other subtypes such as diabetic neuropathy. This contributes to an overestimated prevalence compared with personal health history. A few conditions are, however, of higher prevalence in personal health history compared with EHR data, including obesity, depression, and sleep apnea. Such discrepancies likely reflect a combination of coding biases in EHRs (obesity) and reporting bias in personal health history (depression and sleep apnea).

This study has limitations. Despite the comprehensive approach in the personal health history survey, because of the small sample size per demographic variable (*n* < 20), our analyses were limited to 10 cancer types (*n* = 26 978) and further reduced to 9174 (34%) 5-year survivors for the EHR analysis. This sample size reduction may introduce selection bias and could underestimate the comorbidity burden in the broader cancer population but more likely present long-term cancer survivors. Further, although the All of Us Research Program made notable efforts at recruiting a diverse sample across multiple demographics, participants may not represent the entire US cancer survivor population. Other limitations include surveillance bias, where those receiving follow-up after cancer may receive more comprehensive care. Additionally, the EHR diagnosis date of cancer is uncertain in separating pre- and postcancer diagnosis comorbidity. It is possible that individuals may have an earlier diagnosis that is missing because of change in EHR with fragmented care or a less accessible childhood cancer history. Finally, the comorbidities studied are heterogeneous, with multiple etiologies, and cannot be clearly distinguished from treatment-related toxicity. For example, monoclonal antibody treatment may result in treatment-induced hypertension as opposed to essential hypertension. Further research should consider cancer stage and treatment in relation to comorbidity.

In this large-scale longitudinal analysis, new onset comorbidities affect more than one-half of cancer survivors, with certain cancer types more affected than others. Substantial racial disparities were observed in preexisting and new onset comorbidities. Chronic disease screening tools are needed in cancer survivorship care to monitor comorbidities. Future research is needed to develop effective strategies to prevent new-onset comorbidities during and after cancer treatment.

## Supplementary Material

pkaf093_Supplementary_Data

## Data Availability

The data used in this study are available through the All of Us Research Program’s Researcher Workbench. Access requires registration, identity verification, ethics training, and affiliation with an organization having a Data Use and Registration Agreement. The data can only be analyzed within the secure cloud-based Researcher Workbench environment. Summary statistics and aggregate information are publicly accessible via the All of Us Data Browser (https://databrowser.researchallofus.org).
