# Supplementary material for: Comorbidity prevalence and incidence in cancer survivors: a longitudinal All of Us study
Source: JNCI Cancer Spectr. 2025 Oct 8;9(6):pkaf093. doi: 10.1093/jncics/pkaf093 (PMC12582590; doi:10.1093/jncics/pkaf093)
Supplement: pkaf093_Supplementary_Data [file pkaf093_supplementary_data.docx]

**Supplementary Content**

**Supplemental Tables:**

**Table S1**. Cancer Dictionary from the EHRs in the All-of-Us program.

**Table S2.** Personal Health History Condition List. The survey categorizes conditions into eight major human health systems. Asterisks (*) indicate conditions selected for analysis, based on prevalence within category, observed differences between cancer and non-cancer groups, and clinical relevance and expert knowledge.

**Table S3**. Comparison of sample characteristics between a 34% subset of 5-year survivors and the remaining 66% of the sample (<5-year survivors) in EHR data (N=26,978).

**Table S4.** Repeated analyses of Table 4 were conducted using missing data imputation for the race variable (13.4% missing) and the sex variable (2.77% missing). Quasi-Poisson regression models estimated incidence rate ratio (IRR) for the number of new-onset comorbidities during the five years survival (N = 9,174) by race and age in each cancer type with ten imputed datasets.

**Table S5.** Quasi-Poisson regression models including ethnicity instead of race, as a supplementary analysis to Table 4, estimated the adjusted incidence rate ratio (IRR) for Hispanic or Latino (vs. Not Hispanic or Latino) regarding the number of new-onset comorbidities during the five years of survival (N = 9,174) by each cancer type.

**Table S6**. Analysis using alternative SNOMED codes for neuropathy subtypes in Table3.

**Supplemental Figures:**

**Figure S1**. Flow chart of the study samples from the self-reported personal health history (PHH), N=134,162, in the All-of-Us program.

**Figure S2.** Flow chart of the study cohort from the electronic health record (EHRs), N=26,978, in the All-of-Us program.

**Table S1**. Cancer Dictionary from the EHRs in the All-of-Us program. The All-of-Us program collects EHR data from diverse US healthcare providers, which use different coding systems like ICD9, ICD10, or SNOMED for patient health conditions. The All-of-Us workbench offers a unified search interface from all source vocabularies mapping to the standard concepts. For example, a search keyword, breast cancer, introduced the standard concept group, Malignant tumor of breast (concept code = 254837009) that encompasses 65 standard concept names of participants' conditions. Overall, the search keywords of 11 cancers encompassed 512 standard concept names regarding participants' medical conditions. Once determining the study cohort with 11 cancer conditions (N = 26,978), we furthermore searched for other medical conditions of these individuals. The search keywords were Hypertensive disorder (concept code = 38341003), Osteoarthritis (396275006), Neuropathy (386033004), Depressive disorder (35489007), Obesity (414916001), Type 2 diabetes mellitus (44054006), Sleep apnea (73430006) and Kidney stone (95570007).

| **Cancer Type** | **Standard Concepts Group** | **Standard concept names** | **Source of vocabulary** |
| --- | --- | --- | --- |
| (1) Breast cancer | 254837009  Malignant tumor of breast | Infiltrating duct carcinoma of female breast  Malignant neoplasm of axillary tail of female breast  Malignant neoplasm of female breast  Malignant neoplasm of central part of female breast  Malignant neoplasm of upper-outer quadrant of female breast  Malignant neoplasm of nipple and areola of male breast  Primary malignant neoplasm of breast  Malignant neoplasm of upper-inner quadrant of female breast  Infiltrating duct carcinoma of breast  Malignant neoplasm of lower-outer quadrant of female breast  Primary malignant neoplasm of axillary tail of right female breast  Carcinoma of breast  Infiltrating duct carcinoma of right female breast  Primary malignant neoplasm of axillary tail of breast  Carcinoma of female breast  Malignant neoplasm of lower-inner quadrant of female breast  Local recurrence of malignant tumor of breast  Malignant neoplasm of male breast  Metastatic human epidermal growth factor 2 positive carcinoma of breast  Triple-negative breast cancer  Recurrent primary malignant neoplasm of left female breast  Overlapping malignant neoplasm of male breast  Primary malignant neoplasm of breast lower inner quadrant  Primary malignant neoplasm of skin of breast  Malignant lymphoma of breast  Infiltrating duct carcinoma of left female breast  Malignant melanoma of skin of breast  Adenocarcinoma of breast  Infiltrating lobular carcinoma of breast  Paget's disease of nipple  Infiltrating lobular carcinoma of left female breast  Primary malignant neoplasm of breast lower outer quadrant  Primary malignant neoplasm of female right breast  Malignant neoplasm of breast upper inner quadrant  Malignant melanoma of breast  Primary malignant neoplasm of breast upper inner quadrant  Carcinoma of breast - upper, outer quadrant  Hormone receptor positive malignant neoplasm of breast  Hereditary breast and ovarian cancer syndrome  Mucinous carcinoma of breast  Primary malignant neoplasm of female left breast  Malignant neoplasm of breast upper outer quadrant  Malignant neoplasm of bone, connective tissue, skin and breast  Carcinoma of central portion of breast  Malignant neoplasm of axillary tail of breast  Carcinoma of breast - lower, inner quadrant  Malignant neoplasm, overlapping lesion of breast  Carcinoma of male breast  Sarcoma of female breast  Infiltrating ductal carcinoma of upper outer quadrant of right female breast | SNOMED  ICD9CM  ICD10CM |
| (2) Prostate cancer | 399068003  Malignant tumor of prostate | Prostate cancer metastatic to bone  Malignant tumor of prostate  Primary malignant neoplasm of prostate  Adenocarcinoma of prostate  Recurrent malignant neoplasm of prostate  Hormone refractory prostate cancer  Hormone sensitive prostate cancer  Metastatic castration-resistant prostate cancer  Carcinoma of prostate |  |
| (3) Blood Cancer | **118600007**Malignant lymphoma  OR  **93143009**Leukemia  OR  **109989006**Multiple myeloma | Follicular non-Hodgkin's lymphoma diffuse follicle center sub-type grade 1  Primary central nervous system lymphoma  Hairy cell leukemia (clinical)  Nodular lymphoma of intra-abdominal lymph nodes  Peripheral T-cell lymphoma (clinical)  Malignant lymphoma of spleen  Follicular non-Hodgkin's lymphoma, large cell (clinical)  Follicular non-Hodgkin's lymphoma, small cleaved cell (clinical)  Malignant lymphoma of lymph nodes of axilla AND/OR upper limb  Acute promyelocytic leukemia, FAB M3  Malignant lymphoma of intrathoracic lymph nodes  Hodgkin's disease, nodular sclerosis (clinical)  Nodular lymphoma of extranodal AND/OR solid organ site  Myeloid leukemia  Acute myeloid leukemia with myelodysplasia-related changes  Chronic lymphoid leukemia in remission  Hodgkin's disease of lymph nodes of multiple sites  Nodular lymphoma of lymph nodes of multiple sites  Hodgkin's disease of intrathoracic lymph nodes  Anaplastic large cell lymphoma, ALK negative  Chronic myelomonocytic leukemia  Hodgkin's disease of lymph nodes of head, face AND/OR neck  Hodgkin's disease, nodular sclerosis of extranodal AND/OR solid organ site  Acute leukemia  Plasma cell leukemia  Lymphosarcoma  Leukemic reticuloendotheliosis of extranodal AND/OR solid organ site  Hodgkin's disease in remission  Leukemia  Hodgkin's paragranuloma of lymph nodes of multiple sites  Sézary's disease of extranodal AND/OR solid organ site  Sézary's disease (clinical)  Hodgkin's disease, nodular sclerosis of lymph nodes of multiple sites  Follicular non-Hodgkin's lymphoma, mixed small cleaved cell and large cell (clinical)  Burkitt's tumor of extranodal AND/OR solid organ site  Plasma cell leukemia in relapse  Marginal zone lymphoma  Lymphosarcoma and reticulosarcoma  Diffuse non-Hodgkin's lymphoma  Nodular lymphoma of lymph nodes of inguinal region and lower limb  Mucosa-associated lymphoma  Chronic leukemia  Hodgkin's disease, nodular sclerosis of intrathoracic lymph nodes  Hairy cell leukemia variant  Hodgkin lymphoma, nodular lymphocyte predominance (clinical)  Chronic myeloid leukemia in remission  Hodgkin's disease, mixed cellularity (clinical)  Leukemia in remission  Primary cutaneous CD30+ large T-cell lymphoma  Hodgkin's disease, nodular sclerosis of lymph nodes of head, face AND/OR neck  Malignant lymphoma of intrapelvic lymph nodes  Hodgkin's disease of extranodal AND/OR solid organ site  Reticulosarcoma of lymph nodes of multiple sites  Malignant lymphoma, convoluted cell type  Burkitt's lymphoma of lymph nodes of multiple sites  Mycosis fungoides of intrapelvic lymph nodes  Malignant lymphoma - small lymphocytic  Primary cutaneous T-cell lymphoma  Adult T-cell leukemia/lymphoma  Burkitt's tumor of lymph nodes of head, face AND/OR neck  Nodular lymphoma of intrapelvic lymph nodes  Extranodal NK/T-cell lymphoma, nasal type  Malignant lymphoma, follicular center cell  Subcutaneous panniculitis-like T-cell lymphoma  Classical Hodgkin lymphoma  Acute promyelocytic leukemia, FAB M3, in remission  Extranodal marginal zone B-cell lymphoma of mucosa-associated lymphoid tissue (MALT-lymphoma)  Malignant lymphoma in remission  Malignant lymphoma of lymph nodes of inguinal region AND/OR lower limb  Burkitt's lymphoma (clinical)  Hodgkin's disease, lymphocytic-histiocytic predominance of lymph nodes of head, face AND/OR neck  Kappa light chain myeloma  Relapse multiple myeloma  Hodgkin's disease of lymph nodes of inguinal region AND/OR lower limb  Atypical chronic myeloid leukemia  Mycosis fungoides of lymph nodes of head, face AND/OR neck  Plasma cell leukemia in remission  Hodgkin's disease, nodular sclerosis of lymph nodes of axilla AND/OR upper limb  Large cell anaplastic lymphoma  Marginal zone lymphoma of spleen  Primary cutaneous follicular center B-cell lymphoma  Large cell lymphoma of intrapelvic lymph nodes  Lymphocyte-rich classical Hodgkin lymphoma  Hodgkin's disease, lymphocytic-histiocytic predominance of lymph nodes of multiple sites  Malignant lymphoma - lymphocytic, intermediate differentiation  Nodular lymphoma of lymph nodes of head, face and neck  Follicular mucinosis type mycosis fungoides  Hodgkin's disease of intra-abdominal lymph nodes  Hodgkin's granuloma (clinical)  Reticulosarcoma  Leukemic reticuloendotheliosis of lymph nodes of head, face and neck  Acute monocytic leukemia  Lethal midline granuloma  Chronic monocytic leukemia  Hodgkin's disease of intrapelvic lymph nodes  Lymphosarcoma of lymph nodes of multiple sites  Lymphosarcoma of intra-abdominal lymph nodes  Lymphoid leukemia in remission  Burkitt's lymphoma of intra-abdominal lymph nodes  Nodal marginal zone B-cell lymphoma  Relapsing chronic myeloid leukemia  Diffuse follicle center lymphoma  Reticulosarcoma of lymph nodes of inguinal region and lower limb  Myeloid leukemia in remission  Hodgkin's paragranuloma of intrathoracic lymph nodes  Nodular lymphoma of lymph nodes of axilla and upper limb  Mantle cell lymphoma of spleen  Leukemic reticuloendotheliosis of lymph nodes of multiple sites  Angioimmunoblastic T-cell lymphoma  Acute monocytic/monoblastic leukemia  Chronic leukemia in remission  Anaplastic large T-cell systemic malignant lymphoma  Hodgkin's disease, mixed cellularity of lymph nodes of head, face AND/OR neck  Sézary's disease of lymph nodes of multiple sites  Mycosis fungoides of lymph nodes of inguinal region AND/OR lower limb  Acute lymphoid leukemia relapse  Hodgkin's disease, lymphocytic-histiocytic predominance of intrathoracic lymph nodes  T-cell prolymphocytic leukemia  Lymphosarcoma of intrathoracic lymph nodes  Mycosis fungoides of lymph nodes of multiple sites  Mantle cell lymphoma of lymph nodes of multiple sites  Extramedullary plasmacytoma  Hodgkin's disease of spleen  Diffuse large B-cell lymphoma  Monocytic leukemia  Smoldering myeloma  Primary mediastinal (thymic) large B-cell lymphoma  Hodgkin's disease of lymph nodes of axilla AND/OR upper limb  Follicular low grade B-cell lymphoma  Hodgkin's disease, nodular sclerosis of lymph nodes of inguinal region AND/OR lower limb  Lymphosarcoma of lymph nodes of head, face and neck  Erythroleukemia, FAB M6 in remission  Hodgkin's disease, mixed cellularity of spleen  Hodgkin's disease, lymphocytic depletion of intrathoracic lymph nodes  Splenic marginal zone B-cell lymphoma  Hodgkin's disease, lymphocytic depletion (clinical)  Reticulosarcoma of intra-abdominal lymph nodes  Enteropathy-associated T-cell lymphoma  Subacute myeloid leukemia  Burkitt's lymphoma of intrathoracic lymph nodes  Lymphoid leukemia in relapse  Lymphoma of small bowel  B-cell Hodgkin's lymphoma  Acute myeloid leukemia with t(9:11)(p22;q23); MLLT3-MLL  Burkitt's lymphoma of lymph nodes of inguinal region and lower limb  Hodgkin's disease, lymphocytic-histiocytic predominance of extranodal AND/OR solid organ site  Hodgkin's disease, mixed cellularity of lymph nodes of multiple sites  Leukemic reticuloendotheliosis of intrathoracic lymph nodes  Hodgkin's disease, mixed cellularity of intrathoracic lymph nodes  Erythroleukemia, FAB M6  B-cell lymphoma of lymph nodes of multiple sites  Diffuse non-Hodgkin's lymphoma, lymphoblastic (clinical)  Hepatosplenic T-cell lymphoma  Reticulosarcoma of intrathoracic lymph nodes  Subacute lymphoid leukemia  Mycosis fungoides of spleen  Hodgkin's disease, lymphocytic-histiocytic predominance of intra-abdominal lymph nodes  Burkitt's tumor of lymph nodes of axilla AND/OR upper limb  Malignant lymphoma of thyroid gland  Mycosis fungoides of intra-abdominal lymph nodes  Subacute leukemia  Chronic leukemia in relapse  Nodular lymphoma of intrathoracic lymph nodes  Hodgkin's disease, lymphocytic-histiocytic predominance of lymph nodes of axilla AND/OR upper limb  B-cell prolymphocytic leukemia  Small lymphocytic B-cell lymphoma of intra-abdominal lymph nodes  Large granular lymphocytic leukemia  Hodgkin's disease, lymphocytic depletion of lymph nodes of inguinal region AND/OR lower limb  Acute monocytic leukemia in remission  Megakaryocytic leukemia  Malignant lymphoma of breast  B-cell acute lymphoblastic leukemia  Nodular lymphoma of spleen  Small lymphocytic B-cell lymphoma of lymph nodes of multiple sites  Primary cutaneous B-cell lymphoma  Non-Hodgkin's lymphoma of lymph nodes of multiple sites  Hodgkin's paragranuloma of lymph nodes of head, face AND/OR neck  Subacute myeloid leukemia in remission  Follicular lymphoma, cutaneous follicle centre  Sézary's disease of intra-abdominal lymph nodes  Hodgkin's disease, lymphocytic depletion of intra-abdominal lymph nodes  Gastric lymphoma  Mycosis fungoides of intrathoracic lymph nodes  Mycosis fungoides of lymph nodes of axilla AND/OR upper limb  Hodgkin's disease, nodular sclerosis of intra-abdominal lymph nodes  Sézary's disease of lymph nodes of head, face AND/OR neck  Acute megakaryoblastic leukemia  Subacute monocytic leukemia  Leukemic reticuloendotheliosis of lymph nodes of inguinal region and lower limb  Reticulosarcoma of lymph nodes of head, face and neck  Sézary's disease of lymph nodes of inguinal region AND/OR lower limb  Hodgkin's disease, nodular sclerosis of lymph nodes of head, face and neck  Hodgkin's granuloma of lymph nodes of multiple sites  Burkitt's lymphoma of intrapelvic lymph nodes  Lymphosarcoma of lymph nodes of axilla and upper limb  Sézary's disease of lymph nodes of inguinal region and lower limb  Non-Hodgkin's lymphoma of lung  Hodgkin's disease, mixed cellularity of extranodal AND/OR solid organ site  Hodgkin's disease, lymphocytic-histiocytic predominance of intrapelvic lymph nodes  Hodgkin's disease, lymphocytic depletion of lymph nodes of head, face AND/OR neck  Mast cell leukemia (clinical)  IgG myeloma  IgA myeloma  Hodgkin's disease, lymphocytic-histiocytic predominance of lymph nodes of inguinal region AND/OR lower limb  Leukemic reticuloendotheliosis of lymph nodes of axilla and upper limb  Peripheral T-cell lymphoma - pleomorphic medium and large cell  Hodgkin's disease, lymphocytic-histiocytic predominance of spleen  Hodgkin's paragranuloma of lymph nodes of axilla AND/OR upper limb  Hodgkin's granuloma of lymph nodes of head, face AND/OR neck  Hodgkin's disease, nodular sclerosis of intrapelvic lymph nodes  Diffuse non-Hodgkin's lymphoma, undifferentiated  Diffuse non-Hodgkin's lymphoma undifferentiated (diffuse)  High grade B-cell lymphoma  Acute myelomonocytic leukemia, FAB M4  Malignant lymphoma - mixed small and large cell  Hodgkin's sarcoma of lymph nodes of multiple sites  Relapsing acute myeloid leukemia  Acute panmyelosis with myelofibrosis  Low grade B-cell lymphoma  T-cell lymphoma (clinical)  Hodgkin's granuloma of lymph nodes of inguinal region AND/OR lower limb  Hodgkin's disease, lymphocytic depletion of lymph nodes of multiple sites  Diffuse malignant lymphoma - centroblastic  Hodgkin's paragranuloma of lymph nodes of inguinal region AND/OR lower limb  Therapy related acute myeloid leukemia and myelodysplastic syndrome  Angiocentric NK/T-cell malignant lymphoma involving skin  Burkitt's lymphoma of spleen  Myeloid leukemia in relapse  Peripheral T-cell lymphoma of lymph nodes of multiple sites  Hodgkin's disease, lymphocytic-histiocytic predominance of lymph nodes of head, face and neck  Hodgkin's granuloma of extranodal AND/OR solid organ site  Acute leukemia in remission  Acute lymphoid leukemia in remission  Acute lymphoid leukemia  Acute myeloid leukemia in remission  Acute myeloid leukemia, disease  Chronic lymphoid leukemia, disease  Chronic myeloid leukemia  Malignant lymphoma of intra-abdominal lymph nodes  Malignant lymphoma of lymph nodes of head, face AND/OR neck  Malignant lymphoma of lymph nodes of multiple sites  Malignant lymphoma of extranodal AND/OR solid organ site  Multiple myeloma in remission  Mycosis fungoides of extranodal AND/OR solid organ site  Diffuse non-Hodgkin's lymphoma, small cell (clinical)  Diffuse non-Hodgkin's lymphoma, large cell (clinical)  B-cell lymphoma (clinical)  Multiple myeloma  Hodgkin's disease (clinical)  Malignant lymphoma  Non-Hodgkin's lymphoma (clinical)  Mycosis fungoides (clinical)  Lymphoid leukemia  Waldenström macroglobulinemia  Nodular lymphoma  B-cell chronic lymphocytic leukemia  Cutaneous/peripheral T-cell lymphoma  Follicular non-Hodgkin's lymphoma  Diffuse large B-cell lymphoma (nodal/systemic with skin involvement)  Plasmacytoma  Mantle cell lymphoma  Non-Hodgkin's lymphoma of extranodal site |  |
| (4) Colon Rectal cancer | **781382000**Malignant neoplasm of colon and/or rectum  OR  **363406005**Malignant tumor of colon  OR  **363414004**Malignant tumor of rectosigmoid junction | Primary malignant neoplasm of hepatic flexure of colon  Primary malignant neoplasm of rectum  HNPCC - hereditary nonpolyposis colon cancer  Primary malignant neoplasm of rectosigmoid junction  Primary malignant neoplasm of ascending colon  Primary malignant neoplasm of sigmoid colon  Carcinoma of colon  Primary malignant neoplasm of colon  Malignant tumor of splenic flexure  Malignant tumor of descending colon  Overlapping malignant neoplasm of colon  Primary malignant neoplasm of transverse colon  Malignant tumor of rectosigmoid junction  Malignant carcinoid tumor of colon  Primary malignant neoplasm of descending colon  Malignant tumor of sigmoid colon  Overlapping malignant neoplasm of rectum, anus and anal canal  Malignant tumor of ascending colon  Malignant tumor of rectum  Primary malignant neoplasm of splenic flexure of colon  Adenocarcinoma of rectum  Malignant tumor of colon  Malignant neoplasm of rectum, rectosigmoid junction and anus  Malignant carcinoid tumor of rectum  Malignant tumor of anorectal junction  Malignant tumor of hepatic flexure  Malignant neoplasm, overlapping lesion of colon  Malignant tumor of transverse colon  Primary adenocarcinoma of ascending colon and right flexure  Primary adenocarcinoma of ascending colon  Malignant neoplasm of colon and/or rectum  Carcinoma of transverse colon  Local recurrence of malignant tumor of colon |  |
| (5) Lung cancer | **363358000**  **Malignant tumor of lung** | Primary malignant neoplasm of left upper lobe of lung  Adenocarcinoma of left lung  Primary mucinous adenocarcinoma of lung  Primary malignant neoplasm of middle lobe, bronchus or lung  Primary malignant neoplasm of lower lobe, bronchus or lung  Secondary malignant neoplasm of lung  Malignant neoplasm of lower lobe of lung  Overlapping malignant neoplasm of bronchus and lung  Malignant neoplasm of upper lobe of lung  Malignant tumor of lung  Primary adenocarcinoma of lower lobe of left lung  Secondary malignant neoplasm of left lung  Primary malignant neoplasm of upper lobe, bronchus or lung  Malignant carcinoid tumor of lung  Malignant neoplasm of upper lobe, bronchus or lung  Primary malignant neoplasm of lung  Secondary malignant neoplasm of right lung  Malignant neoplasm of right upper lobe of lung  Malignant neoplasm of upper lobe of left lung  Adenocarcinoma of right lung  Non-small cell lung cancer  Adenocarcinoma of lung  Malignant neoplasm of lower lobe of right lung  Kaposi's sarcoma of lung  Small cell carcinoma of lung  Squamous cell carcinoma of bronchus in left upper lobe  Primary adenocarcinoma of lung  Secondary malignant neoplasm of bilateral lungs  Malignant neoplasm of lower lobe of left lung  Malignant neoplasm of middle lobe, bronchus or lung  Malignant neoplasm of middle lobe of lung  Squamous cell carcinoma of right lung  Primary malignant neoplasm of bronchus of left upper lobe  Squamous non-small cell lung cancer  Metastasis to lung of unknown primary  Squamous cell carcinoma of lung  Non-Hodgkin's lymphoma of lung  Primary malignant neoplasm of lower lobe of left lung  Metastasis to lung from adenocarcinoma  Primary malignant neoplasm of right lung  Carcinoma of lung  Primary malignant neoplasm of bronchus of left lower lobe  Non-small cell carcinoma of lung, TNM stage 4  Non-small cell carcinoma of lung, TNM stage 1  Squamous cell carcinoma of left lung  Primary adenocarcinoma of upper lobe of left lung  Primary adenocarcinoma of upper lobe of right lung  Primary malignant neoplasm of left lung  Malignant tumor of lung parenchyma |  |
| (6) Thyroid cancer | **363478007**  **Malignant tumor of thyroid gland** | Papillary thyroid carcinoma  Primary malignant neoplasm of thyroid gland  Malignant tumor of thyroid gland  Follicular thyroid carcinoma  Carcinoma of thyroid  Malignant lymphoma of thyroid gland  Local recurrence of malignant tumor of thyroid gland  Medullary thyroid carcinoma  Hurthle cell carcinoma of thyroid |  |
| (7) Endometrial (uterus) cancer | **371973000**  **Malignant neoplasm of uterus** | Primary malignant neoplasm of body of uterus  Primary malignant neoplasm of uterine cervix  Malignant neoplasm, overlapping lesion of cervix uteri  Primary malignant neoplasm of endocervix  Primary malignant neoplasm of uterus  Malignant tumor of cervix  Malignant neoplasm of endocervix  Malignant neoplasm of corpus uteri, excluding isthmus  Primary malignant neoplasm of exocervix  Malignant neoplasm of endometrium of corpus uteri  Malignant neoplasm of uterus  Primary malignant neoplasm of endometrium  Endocervical adenocarcinoma  Adenocarcinoma of endometrium  Malignant neoplasm of fundus of corpus uteri  Overlapping malignant neoplasm of body of uterus  Adenocarcinoma of uterus  Primary malignant neoplasm of isthmus of uterus  Adenocarcinoma of cervix  Primary malignant neoplasm of myometrium  Endometrial carcinoma  Malignant neoplasm of body of uterus  Squamous cell carcinoma of cervix  Malignant neoplasm of exocervix  Primary adenocarcinoma of cervix uteri  Malignant neoplasm of isthmus of uterine body  Malignant neoplasm of myometrium of corpus uteri  Sarcoma of endometrium  Adenosquamous carcinoma of cervix  Carcinoma of uterus  Malignant neoplasm of endocervical canal  Carcinoma of endocervix  Sarcoma of uterus  Overlapping malignant neoplasm of uterine cervix |  |
| (8) Kidney cancer | **363518003**  **Malignant tumor of kidney** | Malignant tumor of kidney  Primary malignant neoplasm of kidney  Renal cell carcinoma  Primary malignant neoplasm of renal pelvis  Malignant tumor of renal pelvis  Clear cell carcinoma of kidney  Secondary malignant neoplasm of kidney  Nephroblastoma  Malignant carcinoid tumor of kidney  Renal cell carcinoma of bilateral kidneys  Clear cell carcinoma of right kidney  Transitional cell carcinoma of kidney  Papillary renal cell carcinoma  Malignant tumor of kidney parenchyma  Clear cell carcinoma of left kidney  Transitional cell carcinoma of right kidney  Primary malignant neoplasm of right kidney  Primary malignant neoplasm of left kidney |  |
| (9) Bladder cancer | **399326009**  **Malignant tumor of urinary bladder** | Primary malignant neoplasm of urinary bladder neck  Primary malignant neoplasm of trigone of urinary bladder  Primary malignant neoplasm of anterior wall of urinary bladder  Carcinoma of bladder  Primary malignant neoplasm of bladder  Malignant neoplasm of lateral wall of urinary bladder  Malignant neoplasm, overlapping lesion of bladder  Malignant neoplasm of posterior wall of urinary bladder  Malignant neoplasm of anterior wall of urinary bladder  Secondary malignant neoplasm of bladder  Primary malignant neoplasm of posterior wall of urinary bladder  Malignant tumor of urinary bladder  Primary malignant neoplasm of lateral wall of urinary bladder  Primary malignant neoplasm of dome of urinary bladder  Adenocarcinoma of bladder  Transitional cell carcinoma of bladder  Primary malignant neoplasm of ureteric orifice of urinary bladder  Malignant tumor of trigone of urinary bladder  Malignant tumor of bladder neck  Metastatic malignant neoplasm to dome of urinary bladder  Malignant tumor of ureteric orifice  Malignant tumor of vault of bladder |  |
| (10) Ovarian cancer | **363443007**  **Malignant tumor of ovary** | Primary malignant neoplasm of ovary  Secondary malignant neoplasm of ovary  Primary malignant neoplasm of both ovaries  Secondary malignant neoplasm of right ovary  Malignant tumor of ovary  Secondary malignant neoplasm of left ovary  Clear cell adenocarcinoma of ovary  Malignant epithelial tumor of ovary  Hereditary breast and ovarian cancer syndrome  Primary malignant neoplasm of left ovary  Endometrioid carcinoma ovary  Primary high grade serous adenocarcinoma of ovary  Granulosa cell tumor of ovary  Malignant immature teratoma of ovary |  |
| (11) Cervical cancer | 363354003  Malignant tumor of cervix | Primary malignant neoplasm of uterine cervix  Malignant neoplasm of endocervix  Malignant tumor of cervix  Malignant neoplasm, overlapping lesion of cervix uteri  Primary malignant neoplasm of exocervix  Primary malignant neoplasm of endocervix  Endocervical adenocarcinoma  Adenocarcinoma of cervix  Primary adenocarcinoma of cervix uteri  Malignant neoplasm of exocervix  Squamous cell carcinoma of cervix  Carcinoma of endocervix  Overlapping malignant neoplasm of uterine cervix  Adenosquamous carcinoma of cervix  Malignant neoplasm of endocervical canal |  |

**Table S2.** Personal Health History Condition List. The survey question categorizes conditions into eight major human health systems. Asterisks (*) indicate conditions selected for analysis, based on their prevalence within category and the prevalence differences between cancer vs. non-cancer individuals.

| **Question categories** | **Conditions** | **No Cancer** | **Cancer** |
| --- | --- | --- | --- |
| Circulatory conditions | Anemia | 21509 (12.5%) | 5529 (12.4%) |
|  | Atrial Fibrillation | 5781 (3.4%) | 2446 (5.5%) |
|  | Bleeding Disorder | 1994 (1.2%) | 708 (1.6%) |
|  | Congestive Heart Failure | 2151 (1.2%) | 871 (1.9%) |
|  | Coronary Artery | 4389 (2.5%) | 1747 (3.9%) |
|  | Heart Attack | 2914 (1.7%) | 1072 (2.4%) |
|  | Heart Valve Disease | 2956 (1.7%) | 1170 (2.6%) |
|  | High Cholesterol | 35755 (20.7%) | 9995 (22.4%) |
|  | How Old Were You Allergies: Adult | <20 (0%) | <20 (0%) |
|  | How Old Were You Urinary Tract: Adolescent | <20 (0%) | <20 (0%) |
|  | **Hypertension*** | 33871 (19.6%) | 9604 (21.5%) |
|  | No Heart or Blood Condition | 42178 (24.5%) | 4819 (10.8%) |
|  | Other Heartor Blood Condition | 7877 (4.6%) | 2561 (5.7%) |
|  | Peripheral Vascular Disease | 1377 (0.8%) | 584 (1.3%) |
|  | PMI: Skip | 3012 (1.7%) | 850 (1.9%) |
|  | Pulmonary Embolism | 2518 (1.5%) | 1124 (2.5%) |
|  | Sickle Cell Disease | 188 (0.1%) | 38 (0.1%) |
|  | Stroke | 1915 (1.1%) | 709 (1.6%) |
|  | Transient Ischemic Attack | 2061 (1.2%) | 866 (1.9%) |
| Skeletal Muscular Conditions | Carpal Tunnel | 13139 (8.4%) | 3585 (9.5%) |
|  | Circulatory Conditions: Anemia | <20 (0%) | <20 (0%) |
|  | Fibromyalgia | 5716 (3.7%) | 1432 (3.8%) |
|  | Fractured Broken Bone | 9526 (6.1%) | 2796 (7.4%) |
|  | Gout | 3736 (2.4%) | 1384 (3.7%) |
|  | How Old Were You Urinary Tract: Adolescent | <20 (0%) | <20 (0%) |
|  | No Bone Joint Muscle | 54130 (34.7%) | 6516 (17.3%) |
|  | Osteoarthritis | 20943 (13.4%) | 6727 (17.9%) |
|  | **Osteoporosis*** | 7136 (4.6%) | 3137 (8.4%) |
|  | Other Arthritis | 7654 (4.9%) | 2228 (5.9%) |
|  | Other Bone Joint Muscle | 13166 (8.4%) | 3342 (8.9%) |
|  | PMI: Skip | 2701 (1.7%) | 1073 (2.9%) |
|  | Pseudogout | 327 (0.2%) | 151 (0.4%) |
|  | Rheumatoidarthritis | 5546 (3.6%) | 1524 (4.1%) |
|  | Spine Muscle Bone | 11139 (7.1%) | 3420 (9.1%) |
|  | Systemic Lupus | 1074 (0.7%) | 250 (0.7%) |
| Nervous System Condition | Cerebral Palsy | 158 (0.1%) | <20 (0%) |
|  | Chronic Fatigue | 5540 (3.8%) | 1457 (4.5%) |
|  | Circulatory Conditions: Anemia | <20 (0%) | <20 (0%) |
|  | Concussion | 8722 (6%) | 1954 (6%) |
|  | Dementia | 188 (0.1%) | 111 (0.3%) |
|  | Epilepsy | 2626 (1.8%) | 695 (2.1%) |
|  | How Old Were You Urinary Tract: Adolescent | <20 (0%) | <20 (0%) |
|  | Insomnia | 10063 (6.9%) | 2370 (7.3%) |
|  | Lou Gehrig’s | 37 (0%) | <20 (0%) |
|  | Memory Loss | 3341 (2.3%) | 1129 (3.5%) |
|  | Migraine | 20507 (14.1%) | 3986 (12.3%) |
|  | Multiple Sclerosis | 1083 (0.7%) | 184 (0.6%) |
|  | Muscular Dystrophy | 127 (0.1%) | 31 (0.1%) |
|  | Narcolepsy | 676 (0.5%) | 107 (0.3%) |
|  | **Neuropathy*** | 6714 (4.6%) | 3233 (9.9%) |
|  | No Brain Nervous System | 66798 (45.8%) | 11401 (35.1%) |
|  | No matching concept | <20 (0%) | <20 (0%) |
|  | Other Brain Nervous System | 4151 (2.8%) | 1081 (3.3%) |
|  | Parkinson's Disease | 426 (0.3%) | 148 (0.5%) |
|  | PMI: Skip | 4093 (2.8%) | 1500 (4.6%) |
|  | Restless Leg Syndrome | 6481 (4.4%) | 1865 (5.7%) |
|  | Spinal Cord Injury | 2598 (1.8%) | 836 (2.6%) |
|  | Traumatic Brain Injury | 1611 (1.1%) | 405 (1.2%) |
| Mental Health Condition | ADHD | 7112 (4.3%) | 989 (3.1%) |
|  | Alcohol Use | 3914 (2.4%) | 910 (2.9%) |
|  | Anxiety | 26146 (15.8%) | 4120 (13.1%) |
|  | Autism Spectrum | 788 (0.5%) | 88 (0.3%) |
|  | Bipolar | 4632 (2.8%) | 741 (2.3%) |
|  | **Depression*** | 35317 (21.4%) | 6648 (21.1%) |
|  | Drug Use | 2280 (1.4%) | 413 (1.3%) |
|  | Eating Disorder | 3965 (2.4%) | 613 (1.9%) |
|  | No Mental Health Substance Use | 62151 (37.6%) | 12942 (41%) |
|  | Other Mental Health Substance Use | 1886 (1.1%) | 283 (0.9%) |
|  | Personality Disorder | 1574 (1%) | 249 (0.8%) |
|  | PMI: Skip | 2991 (1.8%) | 1340 (4.2%) |
|  | PTSD | 9067 (5.5%) | 1769 (5.6%) |
|  | Schizophrenia | 597 (0.4%) | 77 (0.2%) |
|  | Social Phobia | 2739 (1.7%) | 364 (1.2%) |
| Other conditions | Acne | 24458 (11.5%) | 4081 (8.7%) |
|  | Allergies | 48152 (22.6%) | 10069 (21.5%) |
|  | Endometriosis | 6087 (2.9%) | 1684 (3.6%) |
|  | Enlarged Prostate | 6297 (3%) | 3017 (6.4%) |
|  | Fibroids | 10372 (4.9%) | 3045 (6.5%) |
|  | **Obesity*** | 26515 (12.4%) | 5505 (11.7%) |
|  | Other Diagnosis | 6368 (3%) | 1373 (2.9%) |
|  | PCOS | 4288 (2%) | 633 (1.4%) |
|  | PMI: Skip | 26280 (12.3%) | 4733 (10.1%) |
|  | Reactions Anesthesia | 2435 (1.1%) | 873 (1.9%) |
|  | Skin Condition | 17833 (8.4%) | 3701 (7.9%) |
|  | Vitamin B Deficiency | 7031 (3.3%) | 1883 (4%) |
|  | Vitamin D Deficiency | 27317 (12.8%) | 6285 (13.4%) |
| Endocrine Conditions | Hyperthyroidism | 3138 (2.6%) | 1159 (4.4%) |
|  | Hypothyroidism | 13335 (10.9%) | 4336 (16.5%) |
|  | No Hormone Endocrine | 73241 (60.1%) | 11181 (42.5%) |
|  | Other Diabetes | 891 (0.7%) | 194 (0.7%) |
|  | Other Hormone Endocrine | 3793 (3.1%) | 981 (3.7%) |
|  | Other Thyroid | 2358 (1.9%) | 977 (3.7%) |
|  | PMI: Skip | 3110 (2.6%) | 1484 (5.6%) |
|  | Pre Diabetes | 10190 (8.4%) | 2632 (10%) |
|  | Type 1 Diabetes | 1530 (1.3%) | 285 (1.1%) |
|  | **Type 2 Diabetes*** | 10277 (8.4%) | 3103 (11.8%) |
| Respiratory Conditions | Asthma | 22064 (18.1%) | 4293 (16.4%) |
|  | Chronic Lung | 2540 (2.1%) | 1181 (4.5%) |
|  | No Lung Condition | 72398 (59.5%) | 12399 (47.4%) |
|  | Other Lung Condition | 4539 (3.7%) | 2573 (9.8%) |
|  | PMI: Skip | 2620 (2.2%) | 1015 (3.9%) |
|  | **Sleep Apnea*** | 17462 (14.4%) | 4723 (18%) |
| Kidney Conditions | Acute Kidney No Dialysis | 965 (0.8%) | 449 (1.9%) |
|  | **Kidney Stones*** | 9503 (8.3%) | 2701 (11.2%) |
|  | Kidney With Dialysis | 455 (0.4%) | 117 (0.5%) |
|  | Kidney Without Dialysis | 2023 (1.8%) | 909 (3.8%) |
|  | No Kidney Condition | 96472 (84%) | 16959 (70.6%) |
|  | Other Kidney Condition | 3104 (2.7%) | 1565 (6.5%) |
|  | PMI: Skip | 2269 (2%) | 1311 (5.5%) |

| **Table S3**. Comparison of sample characteristics between a 34% subset of 5-year survivors and the remaining 66% of the sample (<5-year survivors) in EHR data (N=26,978). | | | | | |
| --- | --- | --- | --- | --- | --- |
| **Variable** | **Category** | **Cancer survivors <5 yrs**  **(N=17,804, 66%)** | | **Cancer survivors 5+ yrs**  **(N=9,174, 34%)** | |
|  |  | **% of N** | **Mean (SD)** | **% of N** | **Mean (SD)** |
| **Age** | 18-39 | 8.5% | 2.22 (2.0) | 7.39% | 2.86 (2) |
|  | 40-54 | 22.87% | 2.89 (2.1) | 27.55% | 3.38 (2) |
|  | 55-64 | 28.09% | 3.13 (2.0) | 33.97% | 3.53 (1.9) |
|  | 65-74 | 28.95% | 3.15 (1.9) | 25.59% | 3.61 (1.8) |
|  | 75+ | 11.59% | 3.11 (1.8) | 5.5% | 3.53 (1.6) |
| **Sex at birth** | Female | 59.51% | 3.02 (2.0) | 59.83% | 3.4 (1.9) |
|  | Male | 37.65% | 2.97 (2.0) | 37.33% | 3.55 (1.9) |
|  | Missing or intersex | 2.84% | 3.13 (2.0) | 2.82% | 3.42 (1.9) |
| **Race** | Asian | 2.11% | 2.07 (1.7) | 1.56% | 2.52 (1.6) |
|  | Black | 15.18% | 3.49 (2.0) | 11.21% | 4.14 (1.8) |
|  | Remaining^a^ | 1.83% | 2.70 (1.9) | 1.68% | 3.56 (2) |
|  | White | 63.52% | 2.87 (2.0) | 72.16% | 3.31 (1.9) |
|  | Missing | 17.36% | 3.2 (2.1) | 13.4% | 3.75(1.9) |
| **Ethnicity** | Hispanic or Latino | 14.09% | 3.18 (2.1) | 9.98% | 3.84 (1.9) |
|  | Not Hispanic or Latino | 81.28% | 2.96 (2.0) | 85.61% | 3.41 (1.9) |
|  | Missing | 4.63% | 3.08 (2.0) | 4.4% | 3.47 (1.9) |
| **^a^** Remaining category includes Middle Eastern or North African, Native Hawaiian or Other Pacific Islander, and More than one population | | | | | |

**Table S4.** Repeated analyses of Table 4 were conducted using missing data imputation for the race variable (13.4% missing) and the sex variable (2.77% missing). Quasi-Poisson regression models estimated incidence rate ratio (IRR) for the number of new-onset comorbidities during the five years survival (N = 9,174) by race and age in each cancer type with ten imputed datasets.**^a^**

| **Cancer Type** | **Mean (SD)**  **Q2 and Q3 ^b^** | **Age** | **IRR**  **(ref: 18-54)** | **95%CI** | **P-value^c^** | **Race** | **IRR**  **(ref: White)** | **95%CI** | **P-value^c^** |
| --- | --- | --- | --- | --- | --- | --- | --- | --- | --- |
| (1) Breast Cancer  (N=3093) | 1.3 (1.4) | 55-64 | 1.14 | (1.02,1.27) | 0.011 | Asian | 0.92 | (0.64,1.33) | 0.89 |
|  | Q2=1, Q3=2 | 65-74 | 1.37 | (1.22,1.54) | <0.001 | Black | 1.59 | (1.37,1.84) | <0.001 |
|  |  | ≥75 | 1.39 | (1.10,1.77) | 0.003 | Remaining**^d^** | 1.45 | (1.07,1.98) | 0.01 |
| (2) Prostate Cancer  (N=1860) | 1.1 (1.2) | 55-64 | 1.28 | (1.04,1.58) | 0.013 | Asian | 1.21 | (0.57,2.57) | 0.85 |
|  | Q2=1, Q3=2 | 65-74 | 1.36 | (1.10,1.68) | 0.002 | Black | 1.34 | (1.12,1.61) | <0.001 |
|  |  | ≥75 | 1.47 | (1.12,1.93) | 0.002 | Remaining**^d^** | 1.44 | (0.96,2.18) | 0.09 |
| (3) Blood Cancer  (N=1545) | 1.4 (1.4) | 55-64 | 0.94 | (0.81,1.08) | 0.60 | Asian | 1.10 | (0.65,1.86) | 0.90 |
|  | Q2=1, Q3=2 | 65-74 | 1.15 | (0.98,1.35) | 0.10 | Black | 1.42 | (1.19,1.7) | <0.001 |
|  |  | ≥75 | 1.08 | (0.80,1.46) | 0.90 | Remaining**^d^** | 1.13 | (0.7,1.82) | 0.80 |
| (4) Colon Rectal Cancer  (N=449) | 1.3 (1.4) | 55-64 | 1.15 | (0.87,1.52) | 0.51 | Asian | 0.94 | (0.33,2.67) | >0.99 |
|  | Q2=1, Q3=2 | 65-74 | 1.38 | (1.02,1.85) | 0.03 | Black | 1.11 | (0.77,1.61) | 0.80 |
|  |  | ≥75 | 1.01 | (0.62,1.65) | >0.99 | Remaining**^d^** | 1.14 | (0.38,3.43) | >0.99 |
| (5) Lung Cancer  (N=235) | 1.4 (1.4) | 55-64 | 1.27 | (0.83,1.97) | 0.40 | Asian | 0.42 | (0.08,2.15) | 0.4 |
|  | Q2=1, Q3=2 | 65-74 | 1.22 | (0.79,1.88) | 0.60 | Black | 1.25 | (0.84,1.87) | 0.4 |
|  |  | ≥75 | 1.06 | (0.52,2.16) | >0.99 | Remaining**^d^** | 1.25 | (0.5,3.17) | 0.9 |
| (6) Thyroid Cancer  (N=708) | 1.1 (1.3) | 55-64 | 1.26 | (1.00,1.59) | 0.04 | Asian | 0.70 | (0.3,1.61) | 0.6 |
|  | Q2=1, Q3=2 | 65-74 | 1.63 | (1.20,2.21) | <0.001 | Black | 1.68 | (1.21,2.34) | <0.001 |
|  |  | ≥75 | 1.13 | (0.54,2.39) | 0.94 | Remaining**^d^** | 1.14 | (0.59,2.21) | 0.9 |
| (7) Endometrial Cancer  (N=411) | 1.4 (1.3) | 55-64 | 1.17 | (0.91,1.51) | 0.30 | Asian | 2.02 | (0.95,4.29) | 0.07 |
|  | Q2=1, Q3=2 | 65-74 | 0.84 | (0.61,1.16) | 0.40 | Black | 1.49 | (1.07,2.08) | 0.01 |
|  |  | ≥75 | 1.59 | (0.91,2.77) | 0.10 | Remaining**^d^** | 1.26 | (0.46,3.43) | 0.88 |
| (8) Kidney Cancer  (N=331) | 1.4 (1.3) | 55-64 | 0.93 | (0.69,1.26) | 0.90 | Asian | 1.09 | (0.27,4.46) | 0.99 |
|  | Q2=1, Q3=2 | 65-74 | 1.00 | (0.72,1.40) | >0.99 | Black | 1.38 | (0.97,1.97) | 0.08 |
|  |  | ≥75 | 1.14 | (0.66,1.94) | 0.90 | Remaining**^d^** | 1.60 | (0.84,3.06) | 0.21 |
| (9) Bladder Cancer  (N <340) | 1.3 (1.3) | 55-64 | 1.39 | (0.95,2.03) | 0.10 | Asian | 0.64 | (0.15,2.67) | 0.8 |
|  | Q2=1, Q3=2 | 65-74 | 1.31 | (0.89,1.92) | 0.20 | Black | 1.53 | (0.87,2.67) | 0.2 |
|  |  | ≥75 | 1.38 | (0.88,2.16) | 0.20 | Remaining**^d^** | 1.00 | (0.43,2.32) | >0.99 |
| (10) Ovarian Cancer  (N<210) | 1.0 (1.1) | 55-64 | 0.99 | (0.65,1.50) | >0.99 | Asian | 1.04 | (0.28,3.83) | >0.99 |
|  | Q2=1, Q3=2 | 65-74 | 1.27 | (0.75,2.14) | 0.60 | Black | 1.52 | (0.79,2.96) | 0.3 |
|  |  | ≥75 | 0.56 | (0.08,3.66) | 0.80 | Remaining**^d^** | 0.68 | (0.11,4.14) | 0.9 |

**^a^** IRRs is the pooled estimates from ten imputed datasets, and adjusted for age, race, sex and the number of pre-existing comorbidities

**^b^** Mean (SD), Q2 = median and Q3 = 75% quartile of the number of new comorbidities during 5yrs survival after the cancer diagnosis.

**^c^** The P-values are adjusted by the Dunnett method to address the multiple testing problem.

**^d^** Remaining category includes Middle Eastern or North African, Native Hawaiian or Other Pacific Islander, and More than one population.

**Table S5.** Quasi-Poisson regression models including ethnicity instead of race, as a supplementary analysis to Table 4, estimated the adjusted incidence rate ratio (IRR) for Hispanic or Latino (vs. Not Hispanic or Latino) regarding the number of new-onset comorbidities during the five years of survival (N = 9,174) by each cancer type.

| **Cancer Type** | **Ethnicity (ref: Not Hispanic or Latino)** | **IRR^a^** | **95%CI** | **p-value** |
| --- | --- | --- | --- | --- |
| Breast Cancer (N=3093) | Hispanic or Latino | 1.43 | (1.27,1.62) | <0.001 |
| Prostate Cancer (N=1860) | Hispanic or Latino | 1.31 | (1.06,1.63) | 0.009 |
| Blood Cancer (N=1545) | Hispanic or Latino | 1.42 | (1.20,1.67) | <0.001 |
| Colon Rectal Cancer (N=449) | Hispanic or Latino | 1.63 | (1.25,2.12) | <0.001 |
| Lung Cancer (N=235) | Hispanic or Latino | 1.04 | (0.58,1.84) | >0.99 |
| Thyroid Cancer (N=708) | Hispanic or Latino | 1.18 | (0.91,1.54) | 0.27 |
| Endometrial Cancer (N=411) | Hispanic or Latino | 1.32 | (1.00,1.74) | 0.05 |
| Kidney Cancer (N=331) | Hispanic or Latino | 1.45 | (0.97,2.15) | 0.07 |
| Bladder Cancer (N<340) | Hispanic or Latino | 0.87 | (0.51,1.50) | 0.80 |
| Ovarian Cancer (N<210) | Hispanic or Latino | 1.47 | (0.91,2.37) | 0.10 |

**^a^** IRRs estimated from Quasi-Poisson regression models adjusted for age, sex and the number of pre-existing comorbidities

**Table S6**. Analysis using alternative SNOMED codes for neuropathy subtypes in Table3.

| **Cancer Type** | **Sample Size** | **Neuropathy**  **(%)** | **Peripheral Neuropathy (%)** | **Polyneuropathy**  **(%)** |
| --- | --- | --- | --- | --- |
| - | - | SNOMED 386033004 | SNOMED 302226006 | SNOMED 42345000 |
| (1) Breast Cancer | 7759 | 20.1 | 18.8 | 3.7 |
| (2) Prostate Cancer | 4838 | 21.7 | 20.2 | 5 |
| (3) Blood Cancer | 4535 | 24.1 | 22.1 | 7 |
| (4) Colon Rectal Cancer | 2228 | 25.1 | 23.7 | 5.4 |
| (5) Lung Cancer | 1436 | 32.7 | 31.1 | 10.2 |
| (6) Thyroid Cancer | 1722 | 24.6 | 22.2 | 3.9 |
| (7) Endometrial Cancer | 1538 | 22 | 20.4 | 4.3 |
| (8) Kidney Cancer | 1205 | 25.1 | 24.1 | 7.6 |
| (9) Bladder Cancer | 975 | 28.7 | 26.9 | 7.4 |
| (10) Ovarian Cancer | 742 | 22.1 | 19.3 | 4.4 |

**Figure S1.** Flow chart of the study samples from the self-reported personal health history (PHH), N=134,162, in the All-of-Us program.

**Figure S2.** Flow chart of the study cohort from the electronic health record (EHRs), N=26,978, in the All-of-Us program.
